# ATM splicing variants as biomarkers for low dose dexamethasone treatment of A-T

**DOI:** 10.1186/s13023-017-0669-2

**Published:** 2017-07-05

**Authors:** Michele Menotta, Sara Biagiotti, Chiara Spapperi, Sara Orazi, Luigia Rossi, Luciana Chessa, Vincenzo Leuzzi, Daniela D’Agnano, Annarosa Soresina, Roberto Micheli, Mauro Magnani

**Affiliations:** 1 Department of Biomolecular Sciences, University of Urbino “Carlo Bo”, 61029 Urbino, PU Italy; 2 Department of Clinical and Molecular Medicine, University “La Sapienza”, 00198 Rome, Italy; 3 Department of Pediatrics and Child Neurology and Psychiatry, University “La Sapienza”, Rome, Italy; 4 Department of Clinical and Experimental Sciences, Pediatrics Clinic and Institute of Molecular Medicine “A. Nocivelli”, Unit of Child Neurology and Psychiatry Spedali Civili and University of Brescia, Brescia, Italy

**Keywords:** Ataxia Telangiectasia, ATM, Dexamethasone, Intra-erythrocyte DEXA, ATMdexa1

## Abstract

**Background:**

Ataxia Telangiectasia (AT) is a rare incurable genetic disease, caused by biallelic mutations in the *Ataxia Telangiectasia-Mutated* (*ATM)* gene. Treatment with glucocorticoid analogues has been shown to improve the neurological symptoms that characterize this syndrome. Nevertheless, the molecular mechanism underlying the glucocorticoid action in AT patients is not yet understood. Recently, we have demonstrated that Dexamethasone treatment may partly restore ATM activity in AT lymphoblastoid cells by a new ATM transcript, namely ATMdexa1.

**Results:**

In the present study, the new ATMdexa1 transcript was also identified in vivo, specifically in the PMBCs of AT patients treated with intra-erythrocyte Dexamethasone (EryDex). In these patients it was also possible to isolate new “ATMdexa1 variants” originating from canonical and non-canonical splicing, each containing the coding sequence for the ATM kinase domain. The expression of the ATMdexa1 transcript family was directly related to treatment and higher expression levels of the transcript in patients’ blood correlated with a positive response to Dexamethasone therapy. Neither untreated AT patients nor untreated healthy volunteers possessed detectable levels of the transcripts. ATMdexa1 transcript expression was found to be elevated 8 days after the drug infusion, while it decreased 21 days after treatment.

**Conclusions:**

For the first time, the expression of ATM splicing variants, similar to those previously observed in vitro, has been found in the PBMCs of patients treated with EryDex. These findings show a correlation between the expression of ATMdexa1 transcripts and the clinical response to low dose dexamethasone administration.

**Electronic supplementary material:**

The online version of this article (doi:10.1186/s13023-017-0669-2) contains supplementary material, which is available to authorized users.

## Background

In the last few years, experimental investigations and clinical trials have provided evidence that short-term treatment with glucocorticoid (GC) analogues is able to improve neurological symptoms in AT patients [1–4] raising the possibility that cerebellar abnormalities may still be modified during the initial phase of cell loss [5]. However, this neurological improvement is transitory and disappears when oral GC administration is discontinued, and the benefit-to-risk balance limits the long-term administration of oral steroids.

The administration of low doses of GC through autologous erythrocytes could reduce steroid toxicity without affecting drug efficacy [6–9] . Indeed, a phase II clinical study IEDAT (EudraCT Number 2010–022315-19, a 6-month single-arm open labeled trial), which envisaged the long-term treatment of AT patients with intra-erythrocyte Dexamethasone (Dexa), has recently been concluded [10]. The treatment led to a significant improvement in neurological symptoms, while at the same time avoiding typical steroid side effects. However, the specific mechanisms underlying the action of dexamethasone in the treatment of the neurodegenerative disease are still under debate [11, 12]. The Ataxia Telangiectasia-Mutated (*ATM)* gene was first described by Savitsky K, et al. [13]. This gene, which codifies for a protein kinase member of the PI3 Kinase-like Kinase (PIKK) family, is transcribed into 27 different mRNAs, 20 alternatively spliced variants and 7 unspliced forms. Eighteen spliced mRNAs putatively encode good proteins, while the remaining 9 mRNA variants appear not to encode good proteins. (Aceview https://www.ncbi.nlm.nih.gov/IEB/Research/Acembly/index.html). In a recent investigation using lymphoblastoid cell lines (LCLs) derived from AT patients as a model, we showed that the action of glucocorticoids may stem from the prompting of a non-canonical splicing event in the ATM mRNA precursor [14]. Specifically, we were able to identify a transcript of 1582 bp called ATMdexa1.

The main aim of the present study was to investigate the possible presence of ATMdexa1 in the blood cells of patients enrolled in the above-mentioned IEDAT clinical trial. In fact, we were not only able to identify the transcript, whose expression depends on drug administration, but also to show that it can act as a blood molecular marker to gauge therapy responsiveness. Interestingly, in treated patients it was also possible to discover several additional ATMdexa1 variants.

## Methods

### Sample collection, RNA extraction and complementary cDNA synthesis

The blood samples were collected from 10 AT patients (6 males and 4 females, average age 9.7 years) enrolled in the IEDAT clinical trial (IEDAT-ERY01–2010) [10], from 6 untreated AT patients (4 females and 2 males, average age 17.5 years) and 6 untreated healthy volunteers (childhood blood samples, unknown gender). For all the treated patients, blood was drawn 21 days after drug administration, and for 2 of those patients, it was also drawn 8 days after treatment administration. Blood samples were also collected from 5 AT participants (4 females and one male, average age 12.1 years) that were treated with EryDex (compassionate use).

Briefly, 3 ml of blood were collected in appropriate vacutainers (Tempus Blood RNA tubes, Life Technologies) and handled and stored according to the manufacturer’s instructions The blood samples were subsequently processed for total RNA extraction using the Tempus spin RNA isolation kit (Life Technologies) as described in the manufacturer’s instructions. A clean-up was performed using the QIAGEN RNA extraction kit to completely eliminate contaminating DNA. For each sample, 500 ng of RNA was employed as a template for cDNA synthesis using the SMARTScribe Reverse Transcriptase (Takara) in the presence of 50 ng of Random hexamers to prime the reaction.

### Quantitative PCR assays

Two qPCR assays were set up for ATMdexa1 estimation. All PCR reactions were carried out using the ABI PRISM 7500 sequence detection system platform (Applied Biosystems). The first assay was based on SYBR green chemistry by SYBR Premix Ex Taq (Tli RNaseH Plus Takara), with a final amount of MgCl_2_ 3.5 mM, and 300 nM of each primer (Forward 5′-ATCTAGATCGGCATTCAGATTCCA-3′ and Reverse 5′-GCAGACCAGCCAATTACTAAAC-3′). The thermal profile optimized to obtain a specific and linear amplification of ATMdexa1 was set for 30 s at 95 °C, followed by 40 cycles of denaturation (for 10 s at 94 °C), annealing (for 20 s at 65 °C) and extension at 72 °C for 40 s. A melting curve analysis of the amplicons was performed at the end of each reaction.

The second analysis consisted of a 5′ exonuclease-based real-time PCR assay performed with the same primers described above with the addition of 100 nM of the ATMdexa1 probe (5′6-FAM ATGCCACTCAGTGGAAGACTCAGAGAA-BHQ1 3′); the PCR reaction was performed using the Hot-Rescue Real-Time PCR Kit–FP (Diatheva). The thermal profile was performed for 30 s at 95 °C, followed by 40 10-s. cycles at 94 °C and annealing/ extension at 72 °C for 60 s. The FKBP5 and DUSP1 gene expression levels were measured using the TaqMan® Gene Expression Assays kit (Life Technologies) according to the manufacturer’s instructions.

Concomitantly with the targets, the cDNA of HPRT1 was amplified using 5′-TATGCTGAGGATTTGGAAAGGGT-3′ and 5′-CCATCACATTGTAGCCCTCT-3′ as upper and lower primers, respectively. In the 5′ exonuclease qPCR, the 5′-JOE-TATGGACAGGACTGAACGTCTTGC-BHQ1–3′ probe was used and the reactions were also achieved in multiplex mode. The relative quantification of the targets was calculated by the ΔCT method [15] (HPRT1 as reference gene) and plotted as 1/2^ΔCT^.

## Results

Following approval by the ethical committees and the patients or their parents, blood samples of 10 out of 22 patients enrolled in the IEDAT clinical study were obtained. Blood collection was carried out at the end of the treatment, precisely 21 days after the sixth and final infusion of intra-erythrocitary Dexamethasone provided in the clinical study.

The qPCR assays were set up for qualitative and quantitative analysis, to identify the mRNA molecule, previously described in lymphoblastoid cell lines. The assays were validated in terms of efficiency and Target to Reference linearity (not shown).

**Fig. 1:**
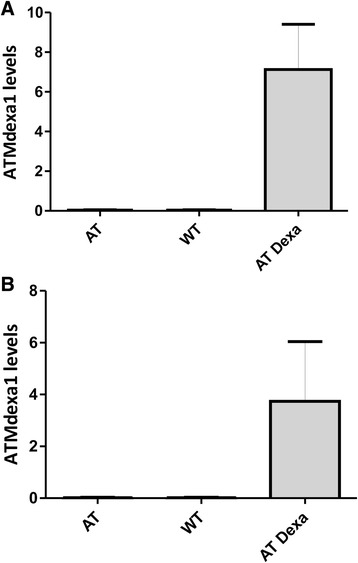
ATMdexa1 expression levels in patients treated with EryDex. Two qPCR assays were set up for ATMdexa1 estimation in whole blood. AT, untreated patients with AT *n* = 6; AT Dexa, patients with AT and treated with EryDex *n* = 10; WT, healthy volunteers *n* = 6. The first qPCR assay is based on SYBR green chemistry PCR (panel **a**) while a second one is built on 5′ exonuclease qPCR (panel **b**). In both settings, ATMdexa1 expression levels were detectable only in AT patients treated with EryDex. The transcript was undetectable in untreated AT patients and in healthy volunteers

ATMdexa1 PCR amplification was carried out in all the 10 IEDAT available samples and also in 6 untreated AT patients and in 6 untreated healthy volunteers. Specific amplification of the target was obtained only in IEDAT samples, while in untreated AT patients and healthy controls, the expression levels were undetectable (Fig. 1a). The amplification carried out by SYBR green PCR on blood samples was similar to those obtained during the assay setup, but a small melting curve deviation was observed among the samples (Additional file 1: Fig. S1). The results obtained using the SYBR green PCR were confirmed by a 5′ nuclease assay as illustrated in Fig. 1b. These results suggest that the induction of the ATMdexa1 RNA messenger is limited to AT patients and strictly dependent on treatment with the drug. The efficacy of EryDex in treating neurological symptoms of AT patients has been evaluated using the ICARS scale [16], and improvements were found to be related to the drug encapsulation efficiency, and therefore to the quantity of the drug administrated [10]. Hence, a direct relationship between drug encapsulation efficiency and ATMdexa1 expression for the 10 available values was tested but the correlation was found to be poor.

**Fig. 2:**
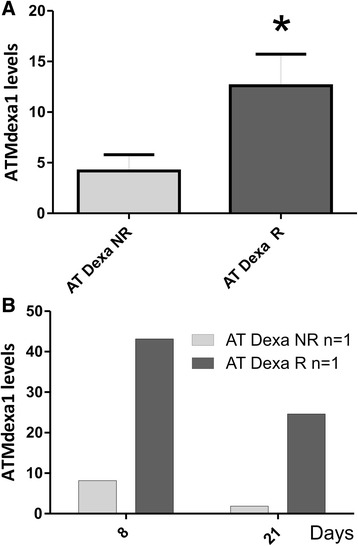
ATMdexa1 expression in responders and non-responders. In panel **a**, AT patients treated with EryDex were divided into two clusters (responders R *n* = 5 and non-responders NR *n* = 5, by average ICARS value decrement of at least 10 points, after six months of therapy). Responders showed higher levels of ATMdexa1 expression (*p* < 0.05) than did non-responders. In panel **b**, for one patient from each cluster, it was possible to evaluate the expression of ATMdexa1 also at +8 days from EryDex administration. In both cases, the extent of the target expression was time dependent

**Fig. 3:**
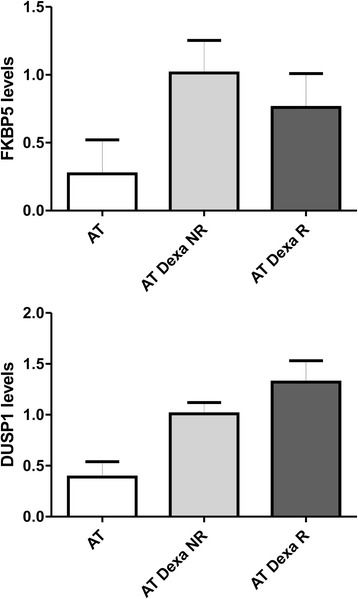
FKBP5 and DUSP1 mRNA expression. Gene expression levels of the two evaluated prototypic targets. AT, untreated patients with AT *n* = 6; AT Dexa, patients with AT and treated with EryDex *n* = 10; WT, healthy volunteers *n* = 6. As predicted, in treated AT patients an improvement in gene expression was observed

In the same previous study by Chessa et al. [10] patients were clustered as either responders or non-responders (AT Dexa R and NR). Responders (ΔICARS ≥ −10) were designated as loaders (Dexa ≥5 mg/bag), while the non-responders (ΔICARS ≤ −9) were designated as poor loaders (Dexa ≤4.9 mg/bag). In the present investigation we were also able to cluster the 10 AT participants into responder (5 AT Dexa R) or non-responder groups (5 AT Dexa NR) using the ΔICARS score. Likewise, the matching ATMdexa1 expression data were assigned to the two clusters. Interestingly, the responders showed a statistically greater ATMdexa1 expression (t-test *p* = 0.0425) than did non-responders (Fig. 2a). Furthermore, for one responder and one non-responder, it was possible to evaluate the ATMdexa1 expression in blood samples collected at +8 and +21 days from EryDex administration. In this case as well the responder showed higher expression levels than those of the non-responder at both times (Fig. 2b). Moreover, by comparing intra-patient levels, it was observed that the ATMdexa1 transcript was expressed at greater levels at +8 day than it was at +21 day. This is consistent with the gradual waning of the DEXA effect over time after treatment, and underlines the need to resort to repeated administrations in order to keep the drug effectiveness active. In order to assess if the improvement in responsiveness depended on glucocorticoid sensitivity the gene expression of two targets (FKBP5 and DUSP1), known to be glucocorticoids modulated [17, 18], were tested (Fig. 3). As expected, the average expression levels of both targets were found to be more elevated than those found in untreated subjects, but not statistically different in the two groups of patients (R and NR).

**Fig. 4:**
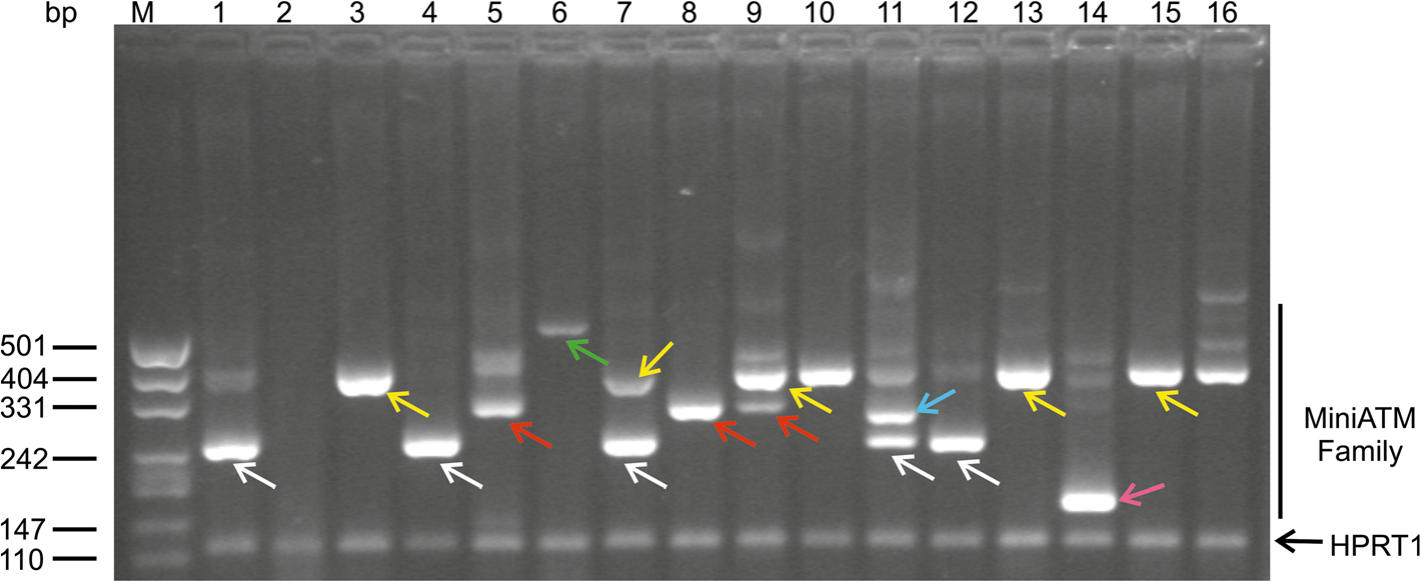
ATMdexa1 transcript family. Amplicons obtained by 5′ nuclease qPCR of the compassionate EryDex samples analysed by electrophoretic gel. Lane M, DNA ladder. Lanes 1–16 contain the qPCR products from five patient samples collected at different time points. The white arrows show the ATMdexa1 PCR product, while the amplicon in all lanes at 115 bp is the HPRT1 PCR product. The other PCR products, with the exception of non-specific amplicons, represent ATMdexa1 splicing variants: green arrow splicing SDR 4–51; yellow arrows splicing 4–53; red arrows splicing 3–52; cyan arrow splicing SDR 3–52 and magenta arrow splicing 2–52

**Fig. 5:**
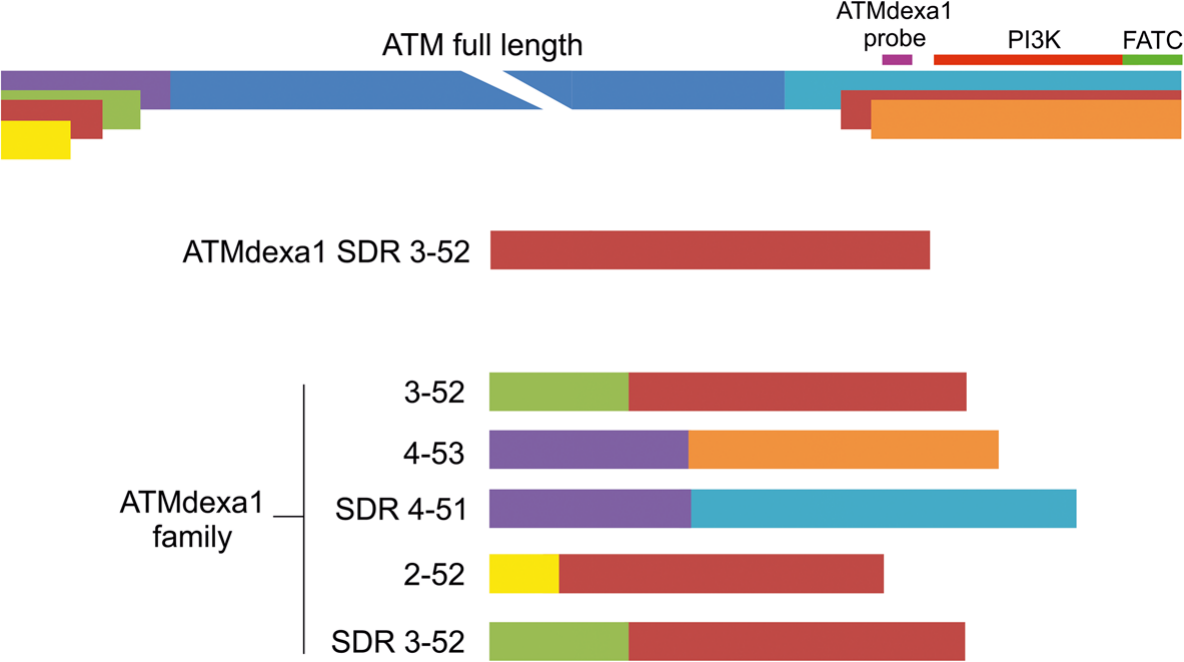
Schematic of the ATMdaxa1 transcripts family. Five ATMdexa1 variants were characterised. Three components of the family derived from canonical splicing while two were from an SDR mediated splicing

The samples from patients treated with EryDex (compassionate usage), allowed a deeper analysis of amplicons obtained by qPCR assays. In these samples the ATMdexa1 was also detected and in order to verify the small temperature deviance observed during the qPCR melting step, as mentioned above, the amplicons were checked by gel electrophoresis. In Fig. 4 lanes 1–16 contain the qPCR products from 5 patient samples collected at different time points. The miniATM PCR product is indicated by white arrows, while the other bands represent unattended amplicons. After the cloning and the DNA sequencing of some of the PCR products, it was possible to define five (currently) new ATM transcripts designated as “ATMdexa1 variants” (Fig. 4 coloured arrows). Every variant contained the qPCR assay probe domain and possessed the coding sequence for the kinase domain of the ATM native protein (Fig. 5). Three of the variants derived from the canonical splicing of the ATM mRNA (exons 3–52, 4–53 and 2–52), whereas two variants originated from a SDR (short direct repeat: 3–52 and 4–51) mediated splicing process. The amplicon size differences from ATMdexa1 ranged from approximately 50 to 160 bp. Some of the described amplicons might explain the observed melting analysis discrepancies during the above-mentioned SYBR green qPCR tests. Samples from dissimilar patients or from the same patients, but at different blood collection times, showed indistinctly variegated ATMdexa1 variants and sometimes even more than one simultaneously (Fig. 4). All the ATMdexa1variants possess the MetMet starting site described by Menotta et al. [14] and can be translated in the miniATM protein by conceptual translation.

## Discussion

In recent years, observational studies and a few clinical trials have shown that short-term treatment of AT patients with oral glucocorticoids is able to improve their neurological symptoms [1–5]. Unfortunately, this improvement is merely transitory and fades shortly after discontinuation of oral treatment with GC. Suspension of the GC treatment is necessary since the hazards long-term therapy with steroids outweigh any potential benefits such a therapy may confer [11]. To overcome this limitation the administration of very low GC doses by erythrocytes reduces steroid toxicity without compromising the drug’s effectiveness [6–9].

In 2010 a phase II clinical study, envisaging the long-term treatment of AT patients by Dexa encapsulated within autologous erythrocytes, was set up [10, 19]. The proposed therapy brought about a significant improvement in neurological symptoms, while avoiding the onset of known side effects typical of GC. Although the mechanism of action of glucocorticoids in AT patients remains unclear, over the past few years several studies have been conducted to shed light on mechanisms underlying the effects of GC in AT patients. D’Assante et al. reported the influence of Betamethasone on molecules involved in autophagosome degradation [20], while the authors of the present study described the influence of Dexamethasone on gene expression, NRF2-mediated antioxidant response by redox balance improvement and cellular nano-mechanics by cytoskeleton and nuclear dynamics [21–23]. Furthermore, despite the observation reported by Savitsky et al. [24] regarding the possibility of alternative splicing of ATM mRNA, we were able to discover and report an alternative splicing event within the coding sequence of the ATM gene product. In fact, we have shown, in lymphoblastoid cell lines established from AT patients, that the action of the synthetic GC Dexamethasone may also be exerted through the assembly of a new mRNA molecule generated by a non-canonical splicing of the *ATM* gene. The resulting transcript, designated as ATMdexa1, has also been identified “in vivo*”* in the blood of AT patients receiving intra-erythrocyte Dexamethasone. The identified splicing procedure is an SDR (short direct repeat) mediated joining, an occurrence described in plants as an environmental stress defense mechanism [25–27]. In mammals, this occurrence has only been described as aberrant and nonfunctional splicing [28, 29] while to our knowledge, the splicing involved in ATMdexa1 assembly represents the only reported case of functional non-canonical SDR splicing.

ATMdexa1 transcript expression levels depend on the treatment with Dexamethasone since, neither untreated AT patients nor untreated healthy volunteers showed detectable levels of the transcript, and its levels are partially correlated with the amount of the administered drug. This correlation was observed in two of the available samples in a time dependent manner. Of considerable interest are the differences in ATMdexa1 expression levels between the two clusters of patients, responders and non-responders. Indeed, on average, responders showed higher levels than non-responders. In light of the fact that the expression of genes such as FKBP5 and DUSP1 are known to be induced by glucocorticoid treatment, ATMdexa1 (miniATM RNA) may be a potential molecular blood marker to gauge treatment efficacy and predict outcome in AT patients. Additional studies with larger sample pools are needed to confirm this hypothesis.

The analysis of the transcripts in additional samples from patients undergoing compassionate treatment with EryDex [19] revealed the existence of not only ATMdexa1 but also of an ATMdexa1 transcript family, detectable by the designed qPCR assays. Unlike the known transcript (ATMdexa1), the transcripts of the new family are formed also by a canonical splicing occurrence.

It is possible to speculate that all these shortened ATM transcripts are latent in human genomics [30] and that Dexa may simply turn on a switch in AT patients to activate a rescue program by multiple ATMdexa1 transcripts potentially translatable into miniATM proteins. The probability of latent and inducible splicing has been extensively investigated in genomic studies [31], and the use of exon skipping is an established strategy in some severe diseases such as Duchenne muscular dystrophy [32]. Currently we have no data on the presence of these shorter transcripts in other cell lines or tissues, but the detection of the ATMdexa1 family in vivo suggests that a similar response may occur in other organs.

## Conclusions

This investigation has shown that the ATMdexa1 transcript is detectable in the whole blood of AT patients treated with EryDex. The expression levels of the transcript were found to depend on drug administration and time from drug infusion, and to correlate with the patients’ responsiveness to treatment. Hence, the detection of the ATMdexa1 transcript may represent a molecular marker that could be used as a prognostic marker to gauge drug effectiveness. Additional clinical trials are needed to further test these hypotheses. In the present study we also describe for the first time the presence of an ATMdexa1 transcript family composed of multiple splicing forms of ATM mRNA, similar to ATMdexa1, and potentially translatable in the miniATM protein. These findings call for additional studies on the role of the multiplicity molecular forms of ATM transcripts induced by Dexamethasone in Ataxia Telangiectasia patients.

## Additional file

Additional file 1: Figure S1. Melting analysis of IEDAT qPCR amplicons. During SYBR green qPCR of the IEDAT samples, although only one PCR product was observable by melting step at the end of the experimental, some Tm discrepancies were noted among the samples. A different kind of ATMdexa1 target was probably amplified without affecting the PCR performance. (TIFF 41 kb)

## Abbreviations

AT: Ataxia Telangiectasia
DEXA: Dexamethasone
EryDex©: Intra-erythrocitary Dexamethasone
GC: Glucocorticoid
