# Supplementary figures and images for: ATM splicing variants as biomarkers for low dose dexamethasone treatment of A-T
Source: Orphanet J Rare Dis. 2017 Jul 5;12:126. doi: 10.1186/s13023-017-0669-2 (PMC5498894; doi:10.1186/s13023-017-0669-2)

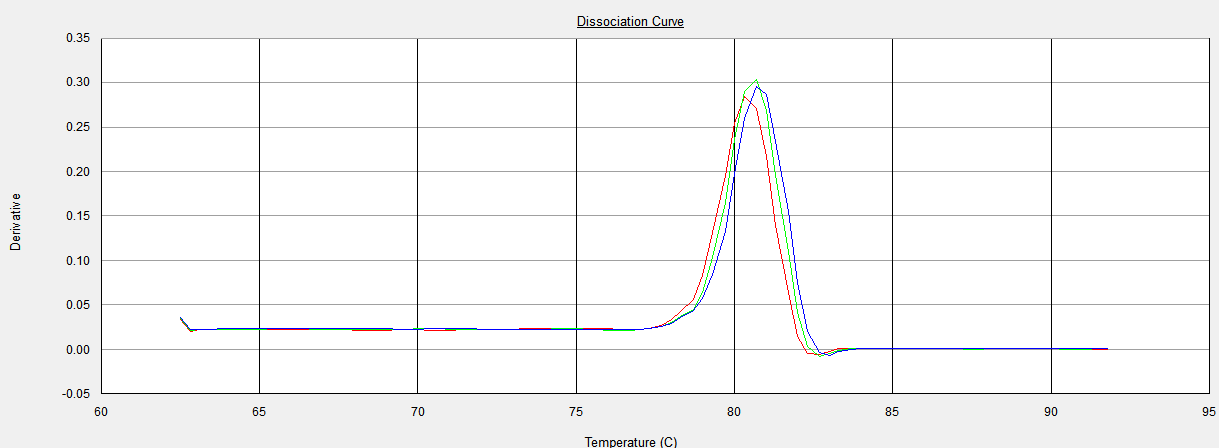

Supplement: Additional file 1: Figure S1. — Melting analysis of IEDAT qPCR amplicons. During SYBR green qPCR of the IEDAT samples, although only one PCR product was observable by melting step at the end of the experimental, some Tm discrepancies were noted among the samples. A different kind of ATMdexa1 target was probably amplified without affecting the PCR performance. (TIFF 41 kb) [file 13023_2017_669_MOESM1_ESM.tif]
